# Uterus preserving surgery versus hysterectomy in the treatment of refractory postpartum haemorrhage in two tertiary maternity units in Cameroon: a cohort analysis of perioperative outcomes

**DOI:** 10.1186/s12884-017-1346-0

**Published:** 2017-05-30

**Authors:** Julius Sama Dohbit, Pascal Foumane, Elie Nkwabong, Christelle Ogolong Kamouko, Joel Noutakdie Tochie, Bernard Otabela, Emile Mboudou

**Affiliations:** 1 Department of Obstetrics and Gynaecology, Gynaeco-Obstetric and Paediatric Hospital, Yaounde, Cameroon; 2 Department of Obstetrics and Gynaecology, Faculty of Medicine and Biomedical Sciences, University of Yaounde I, Yaounde, Cameroon; 3 Department of Obstetrics and Gynaecology, University Hospital Centre, Yaounde, Cameroon; 4 Department of Surgery and sub-Specialties, Faculty of Medicine and Biomedical Sciences, University of Yaounde I, Yaounde, Cameroon; 5 Health and Human development (2HD) Research Group, Douala, Littoral Region Cameroon; 6 Higher Institute of Medical Technologies, Yaounde, Cameroon; 7 Department of Obstetrics and Gynaecology, Gynaeco-Obstetric and Paediatric Hospital, Douala, Cameroon

**Keywords:** Postpartum haemorrhage, Uterus preserving surgery, Hysterectomy, Perioperative outcomes, Cameroon

## Abstract

**Background:**

Little evidence exists on the efficacy and safety of the different surgical techniques used in the treatment of postpartum haemorrhage (PPH). We aimed to compare uterus preserving surgery (UPS) versus hysterectomy for refractory PPH in terms of perioperative outcomes in a sub-Saharan African country with a known high maternal mortality ratio due to PPH.

**Methods:**

This was a retrospective cohort study comparing the perioperative outcomes of all women managed by UPS (defined as surgical interventions geared at achieving haemostasis while conserving the uterus) versus hysterectomy (defined as surgical resection of the uterus to achieve haemostasis) for PPH refractory to standard medical management in two tertiary hospitals in Cameroon from January 2004 to December 2014. We excluded patients who underwent hysterectomy after failure of UPS. Comparison was done using the Chi-square test or Fisher exact test where appropriate. Bonferroni adjustment of the *p*-value was performed in order to reduce the chance of obtaining false-positive results.

**Results:**

We included 24 cases of UPS against 36 cases of hysterectomy. The indications of surgery were dominated by uterine rupture and uterine atony in both groups. Types of UPS performed were seven bilateral hypogastric artery ligations, seven hysterorraphies, six bilateral uterine artery ligations, three B-Lynch sutures and one Tsirulnikov triple ligation with an overall uterine salvage rate of 83.3%. Types of hysterectomies were 26 subtotal hysterectomies and 10 total hysterectomies. UPS was associated with maternal deaths (RR: 2.3; 95% CI: 1.38–3.93.; p: 0.0015) and postoperative infections (RR: 1.96; 95% CI: 1.1–3.49; p: 0.0215). The association of UPS with maternal death was not attenuated after Bonferroni correction. Hysterectomy had no statistically significant adverse outcome.

**Conclusion:**

Hysterectomy is safer than UPS in the management of intractable PPH in our setting. The choice of UPS as first-line surgical management of PPH in resource-limited settings should entail diligent anticipation of these adverse maternal outcomes in order to lessen the perioperative burden of PPH.

## Background

Evidence from a recent systematic review suggests that postpartum haemorrhage (PPH) is the leading cause of maternal mortality worldwide, claiming 480,000 global maternal deaths between 2003 to 2009, of which 41.6% of these PPH-related deaths occurred in sub-Saharan Africa (SSA) [1]. Likewise, in Cameroon, a SSA country, much efforts still need to be done to reduce the current maternal mortality ratio (MMR) from 596 per 100, 000 live births to the targeted global MMR of less than 70 per 100,000 live births by the year 2030 [2]. The way forward partly entails tackling PPH which has been reported as the primary cause of maternal deaths in several recent hospital-based audit reports of the country [3, 4]. In this resource-challenged setting, a composite of factors further contribute to the burden of maternal mortality and include: inadequate attendance of antenatal care [3], poverty [4] and late hospital referral [4]. Efforts to curb PPH-related maternal mortality have targeted various medical measures, non-medical measures, uterus preserving surgical interventions or hysterectomy [5].

Historically, peripartum hysterectomy has been the ultimate surgical management reserved for intractable PPH associated with haemodynamic instability. However, this radical surgery is associated with an inability to carry a future pregnancy and thus, considerable psychological trauma [6, 7]. In order to preserve the uterus for subsequent pregnancies, various uterus preserving surgeries were proposed and consist of either selective ligation of pelvic arteries [8–10] or uterine compression suturing [11]. There are indisputable valid ethical issues hindering the conduct of a randomized controlled trial comparing the efficacy and safety of uterus preserving surgery (UPS) to hysterectomy as first-line surgical management of refractory PPH. Consequently, the highest level of evidence stems from pooled case series and case reports without control groups, carried out in high-income countries suggesting 62 to 100% success rates for various uterus preserving surgical procedures in averting hysterectomy [5]. Although this pooled evidence is low, WHO guidelines recommend UPS as the first-line surgical option in view of its “preserved” reproductive capacity [5]. Meanwhile, other publications mainly in the form of case reports have discussed the cons of UPS for PPH, namely postoperative pyometrium [12], uterine necrosis warranting hysterectomy [13, 14], uterine rupture during subsequent pregnancies [15] and secondary infertility due to postoperative uterine synechia and pelvic adhesions [16, 17]. Hence, we proposed this study to compare the perioperative outcomes of UPS versus hysterectomy for PPH in a selected sub-Saharan African population with a known very high MMR due to PPH.

## Methods

### Study design, setting and participants

This was a cohort study which retrospectively enrolled all women with a minimum gestational age of 28 weeks who underwent first-line surgical management by either UPS or hysterectomy for refractory postpartum haemorrhage following vaginal or caesarean delivery between January 1, 2004 to December 31, 2014 in two university teaching hospitals of Cameroon; the Yaounde Gynaeco-Obstetric and Paediatric Hospital, and the University Hospital Centre of Yaounde. We excluded patients with persistent bleeding managed by another UPS or hysterectomy after initial failure of a UPS. Patients with incomplete medical records were also excluded.

### Definition of terms

PPH was defined as an estimated blood loss greater than 500 ml within 24 h after vaginal delivery and greater than 1000 ml following caesarean section [18]. Refractory or intractable PPH was defined as persistent PPH despite standard medical management (oxytocin, methyl-ergometrine, misoprostol), non-medical management (uterine massage, bimanual uterine compression and then compression of the abdominal aorta), repair of vaginal or cervical lacerations and manual uterine revision where appropriate [5]. Uterus preserving surgery was defined as any surgical intervention consisting of ligation of pelvic arteries or application of uterine compression sutures to achieve haemostasis while concomitantly conserving the uterus e.g. Bilateral hypogastric artery ligation, Uterine artery ligation, B-lynch uterine compression suture, Tsirulnikov triples ligation and Hysterorraphy. Tsirulnikov Triple ligation entailed bilateral ligation of the round ligaments, utero-ovarian ligaments and uterine arteries [19]**.** B-lynch uterine compression suture consisted of making a lower segment transverse hysterotomy or removing the sutures of a recent caesarean section to apply lateral uterine brace sutures to envelop and compress the bleeding uterus in order to achieve haemostasis [11]. Hysterectomy was defined as a surgical procedure geared at achieving haemostasis through resection of the uterus e.g. subtotal abdominal hysterectomy or total abdominal hysterectomy. Total abdominal hysterectomy consisted of complete resection of the uterus and cervix, while the cervical stump was left in-situ in subtotal abdominal hysterectomy.

### Management of postpartum haemorrhage

Uniform and standard operating protocols for the management of PPH were in use in both study settings [5]. Noteworthy, sulprostone was not incorporated in the medical management of PPH due to its non-availability in Cameroon at the time of the study. The decision on whether to perform UPS or hysterectomy as the first surgical management was guided by each patient’s clinical condition. Generally, when the patient presented with haemorrhagic shock, especially if she had four living children, hysterectomy was preferred over UPS. All surgeries were performed by four Consultant Obstetricians (JSD, PF, EN and EM) with more than 10 years of clinical experience after qualifying. Antibiotic prophylaxis was administered intravenously before surgery. Also, all surgical procedures were performed under general anaesthesia and via laparotomy.

### Data collection, variables and measurements

The following study variables were retrieved from patients’ medical records and postoperative notes; (i) Socio-demographic data: age, employment status, marital status and level of education. (ii) Pre-operative characteristics: gestational age, gravid formula, estimated blood loss, and haemodynamic parameters. (iii) Surgical management: type of UPS or hysterectomy and their indication. (iv) Intra-operative complications: intra-operative blood lost, ureteral injuries and maternal death. (v) Postoperative course till discharge: recurrence of haemorrhage, further blood transfusion, surgical site infection, length of hospital stay and maternal death.

### Data management and statistical analysis

Data analysis was performed with Epi Info 3.5.1 software. Distribution of perioperative characteristics were compared between women managed by UPS and those managed by hysterectomy using the Chi-square test or Fisher exact test where appropriate. Relative risk (RR) and their corresponding 95% confidence intervals (95% CI) were calculated in order to measure associations. The original alpha-value was set at 0.05. In order to reduce the chance of obtaining false-positive results from the multiple analyses performed on the same dependent variable, the Bonferroni adjusted *p*-value was calculated by dividing the alpha-value by the number of comparisons. Hence, any comparison was statistically significant if it was inferior to the Bonferroni adjusted *p*-value. Variables with too much missing data precluding meaningful analyses were excluded.

### Ethics consideration

The study was approved by the Institutional Review Board of the Faculty of Medicine and Biomedical Sciences, University of Yaounde I, Yaounde, Cameroon under the ethical clearance No 168/CIERSH/DM/2015. Administrative authorizations were equally obtained from the directorate of both hospitals involved prior to the beginning of the study.

## Results

From January 2004 to December 2014, there were 42,944 deliveries for 1457 cases of PPH, corresponding to an incidence of PPH of 34 per 1000 deliveries. Likewise, 74 cases of PPH managed surgically were recorded, corresponding to an incidence of surgical management of PPH of 1.7 per 1000 deliveries. Of the 74 PPH managed surgically, eight were excluded because they were managed by hysterectomy after failed UPS. Two hospital files were incomplete while four were missing due to an inadequate record-keeping system. Thus we retained 60 eligible cases of surgical management of PPH; 24 uterus preserving surgeries and 36 hysterectomies. Their mean maternal age was 32.6 ± 5.7 years. In both groups, majority were married (71.6%), unemployed (53.3%), multigravidae (90%), multiparous (78.3%), had a level of higher education (56.6%), were referred from another health facility (61.6%) and were at a term pregnancy (80%). The average number of antenatal care consultations of the entire study population was 2.1 ± 1.8.

### Indications for surgical management of PPH

The main indications for both UPS and hysterectomy were uterine rupture, uterine atony and coagulopathy as depicted in Table 1.

**Table 1:** Indications for surgical management of postpartum hemorrhage

Surgical indications	Uterus preserving surgery (*n* = 24)	Hysterectomy (*n* = 36)
Uterine rupture	10 (41.7%)	12 (33.3%)
Uterine atony	10 (41.7%)	11 (30.6%)
Disseminated intravascular coagulation	03 (12.5%)	05 (13.9%)
Placenta abruption	01 (4.1%)	04 (11.1%)
Placenta accreta	00	04 (11.1%)

### Types of surgeries

The types of uterus preserving surgical interventions and their corresponding success rates in preventing hysterectomy are shown in Table 2. On the other hand, the types of hysterectomies performed were 26 (72.2%) subtotal hysterectomies and 10 (27.8%) total hysterectomies.

**Table 2:** Types of uterus preserving surgeries

Types of uterus preserving surgeries	Number (%)	Uterine salvage rate
Bilateral ligation of the hypogastric arteries	07 (29.2)	06 (86%)
Hysterorraphy	07 (29.2)	06 (86%)
Bilateral ligations of the uterine arteries	06 (25)	04 (66.7%)
B-lynch uterine compression suturing	03 (12.5)	03 (100%)
Tsirulnikov triple ligation	01 (4.1)	01 (100%)
Total	24 (100)	20 (83.3%)

### Perioperative complications

UPS was statistically significantly associated with maternal deaths (RR: 2.3; 95% CI: 1.38–3.93.; p: 0.0015) and postoperative infections, mainly endometritis (RR: 1.96; 95% CI: 1.11–3.49; p: 0.0215). The association of UPS with maternal death was not attenuated after Bonferroni adjustment. All cases of maternal death were related to late referral of patients in a state of haemorrhagic shock. Hysterectomy was not significantly associated with an adverse perioperative outcome (Table 3).

**Table 3:** Complications of uterus preserving surgery versus hysterectomy

Perioperative Complications	Uterus preserving surgery (*n* = 24)	Hysterectomy (*n* = 36)	RR	95% CI	*p*-value
Total intraoperative blood lost					
≤1500 ml	14 (58.3%)	22 (61.1%)	0.93	0.49–1.75	0.829
>1500 ml	10 (41.7%)	14 (38.9%)			
Ureteral lesions					
Yes	00	05 (13.9%)	0.19	0.01–2.75	0.223
No	24 (100%)	31 (86.1%)			
Intraoperative cardiac arrest					
Yes	02 (8.3%)	04 (11.1%)	0.82	0.25–2.65	0.738
No	22 (91.7%)	32 (88.9%)			
Postoperative infections					
Yes	10 (41.7%)	06 (16.7%)	1.96	1.11–3.49	0.0215
No	14 (58.3%)	30 (83.3%)			
Maternal deaths					
Yes	07 (29.1%)	02 (5.6%)	2.3	1.38–3.93	0.0015^*^
No	17 (70.8%)	34 (94.4%)			
Length of hospital stay					
≤ 5 days	04 (16.7%)	12 (33.3%)	0.55	0.22–1.36	0.197
> 5 days	20 (83.3%)	24 (66.7%)			

^*^Bonferroni corrected *p*-value <0.0083. RR: Relative Risk; 95% CI: 95% Confidence interval

## Discussion

This study aimed at comparing UPS versus hysterectomy for refractory PPH in terms perioperative outcomes in Cameroon, a sub-Saharan African country with a high maternal mortality ratio due to PPH. We found that uterus preserving surgical management of PPH doubled the risk of maternal mortality and post-operative infections. Furthermore, the association of UPS with maternal death was not attenuated once adjustment for potential false-positive results was made.

Over the 11-year review period, we found an incidence of 1.7 per 1000 deliveries for surgical management of PPH close to the 2.3 per 1000 deliveries reported in France in 2011 [20]. The overall incidence of peripartum hysterectomy in our series was 0.8 per 1000 deliveries, less than the 4 per 1000 deliveries observed by in Ghana [21] and the 3.78 per 1000 deliveries in neighbouring Nigeria [22]. However, this incidence was higher than the 0.48–0.53 per 1000 deliveries reported in high-income countries [23, 24]. The incidence of peripartum hysterectomy varies across the world and it is influenced by the socioeconomic status, standard of obstetric care, cultural values and the acceptability of family planning [25]. In our cohort, the low incidence may be explained by the prevailing local culture which favours fertility and resents hysterectomy. The incidence of conservative surgery was 0.6 per 1000 deliveries, in contrast to the 1.96 per 1000 deliveries reported in France [20], probably due to the fact that UPS for PPH is not yet an integral first-line surgical management of PPH in our setting.

Like prior reports on either UPS [26, 27] or hysterectomy [21, 22] for PPH, we found uterine rupture and uterine atony to be the main indications for surgical management of PPH. In modern obstetrics, abnormal placentation (placenta accreta or placenta praevia) has replaced surgical indications like uterine atony or uterine rupture, because of the increased caesarean section rates, the improved medical management of uterine atony, a reduced incidence of uterine rupture owing to the preference of the lower uterine segment incision over the upper uterine segment incision for caesarean section [24, 28]. Hence, this finding may imply inadequate obstetrical care in our setting with resultant higher complications of uterine rupture or atony. Contributing risk factors to the high frequency of uterine rupture and uterine atony in our cohort include the high proportion of multiparous women (78.3%), a known risk factor of PPH [29] and peripartum hysterectomy [23]. This highlights the need for an adequate family planning policy in these two hospitals. Noteworthy, previously reported risk factors for the high incidence of PPH in Cameroon [3, 4] were prevalent in our study population as follows; poor antenatal care attendance (evident by a mean number antenatal consultations of two) and unemployment (53.3%). These findings pinpoint the deleterious effects of financial constraints on the accessibility to healthcare during pregnancy in this country. Hence, the subsequent risk of unanticipated PPH. The formulation of health policies targeting these risk factors for PPH cannot be overemphasized.

The types of UPS performed were seven bilateral hypogastric artery ligation, seven hysterorraphies, six bilateral uterine artery ligations, three B-Lynch uterine compressive suturing and one Tsirulnikov triple ligation. The uterine salvage rate for bilateral hypogastric artery ligation was 86%, higher than previously reported rates of 42–75% [30, 31]. We observed a uterine salvage rate of 66.7% for uterine artery ligation which was inferior to prior reports of 80–96.2% [32, 33]. Meanwhile the success rates for uterine compressive suturing and Tsirulnikov triple ligation (100%) were similar to previously published data [11, 19, 34]. Bilateral hypogastric artery ligation is the oldest known UPS, first described in 1960 by Sagarra et al. [8]. This may explain the reason why there was a greater resort to this procedure by obstetricians compared to the B-Lynch compressive suturing which is the most recent in the armamentarium of UPS for PPH. With regards to hysterectomy, subtotal hysterectomy and total abdominal hysterectomies were performed in 26 and 10 cases, respectively. There is often a therapeutic debate on the benefits of subtotal versus total abdominal hysterectomy [25, 35]. Our predilection for the former was due to its technical ease with shorter operative time, less blood loss and reduced risk of urologic injuries in an emergency situation with haemorrhagic shock. However, some authors observed similar outcomes for both types of hysterectomies [25, 28].

UPS was associated with postoperative endometritis (RR: 1.96; 95% CI: 1.11–3.49; p: 0.0215), notably B-Lynch compressive sutures explained by the fact that this surgical technique entails a hysterotomy and endo-uterine manipulations which are known risk factors for endometritis. Contrary to past concepts [36, 37], in our study, maternal deaths were more frequent in UPS compared to hysterectomy (29.1% vs. 5.6%; RR: 2.3; 95% CI: 1.38–3.93.; p: 0.0015). We attribute this to delay in deciding to perform UPS, the lack of a decisional clinical algorithm, hypovolemic shock and the irregular supply of blood products and oxygen for appropriate resuscitation in our resource-limited setting. Moreover, the absence of a national health insurance policy was a contributing factor to maternal mortality in the uterus preserving surgical group because management was delayed in three cases of maternal deaths where the patient’s family could not immediately afford to pay for the cost of healthcare.

With extensive literature search, to our knowledge, this study is one of the first comparative studies on UPS versus hysterectomy for refractory PPH in sub-Saharan Africa. Its strength lies in its cohort design over a wide review period of 11 years to assess this comparison. Its findings may guide obstetricians in making informed decisions on the various types of surgical techniques for the management of intractable PPH in resource-limited settings. Other similarly large studies were case series on the outcomes of UPS alone [10, 26, 27, 32, 36] or hysterectomy alone [21, 22]. Its main limitation is the inability to assess the surgical expertise of the operating obstetricians, which is a paramount determinant of the success rates of the type of surgical intervention undertaken. However, all the obstetricians who performed either the UPS or hysterectomy in this cohort had a minimum of 10 years of clinical experience after qualifying and were familiar with all the surgical procedures performed. Also this study was not designed to identify long-term complications of UPS such as secondary infertility or uterine rupture during subsequent pregnancies, highlighting the need for further research in this domain.

## Conclusions

This is one of the largest and first series comparing the perioperative outcomes of uterus preserving surgery versus hysterectomy for refractory PPH in Cameroon and perhaps sub-Saharan Africa at large. Its results suggest higher perioperative mortality for uterus preserving surgery than hysterectomy in this resource-limited environment, which persist even after adjusting for potential false-positive results. Hence, the choice of uterus preserving surgery over hysterectomy as first-line surgical management of intractable PPH in resource-limited settings should entail diligent anticipation of the aforementioned adverse maternal outcomes and vigorous scrutiny of the available health infrastructure in order to lessen the perioperative burden of PPH.

## Abbreviations

MDG: Millennium development goal
MMR: Maternal mortality ratio
PPH: Postpartum haemorrhage
SSA: Sub-Saharan Africa
UPS: Uterus preserving surgery
